# Supplementary material for: Effectiveness of a positive deviance approach to improve appropriate feeding and nutritional status in South West Region, Ethiopia: A study protocol for a cluster randomized control trial
Source: PLoS One. 2024 Jan 2;19(1):e0266151. doi: 10.1371/journal.pone.0266151 (PMC10760826; doi:10.1371/journal.pone.0266151)
Supplement: S2 File — (DOCX) [file pone.0266151.s002.docx]

**Title :** Effectiveness of a positive deviance approach to improve appropriate feeding and nutritional outcomes in South West Region, Ethiopia: A study protocol for a cluster randomized control trial

Principal Investigator: Abraham Tamirat Gizaw

ABSTRACT

**Background:** Non-optimal infant and young child feeding (IYCF) practices are associated with malnutrition, infant mortality and under-five mortality, especially in Sub-Saharan Africa, and particularly in Ethiopia. Most growth faltering in developing countries occurs in the first two years of life; therefore, there is a need to identify interventions for vulnerable communities that improve child nutritional status during this critical period. Positive deviance inquiry is effective in identifying advantageous health behaviors and improving health outcomes among disadvantaged resource-poor populations. This project elucidates the effectiveness of the positive deviant approach in tackling child malnutrition as compared with other interventions done previously in Ethiopia related to nutritional interventions. Although the long-term sustainability of nutrition programs may require something more tangible than simply good behavioral-change communication, we need interventions reveals at least partial solutions today to difficult problems, thereby permitting some action now and building enthusiasm for the long haul that encourage the community engagement and participation to solve their problem by themselves. Therefore, the finding of this study will provide future directions for policy and practice targeting to improve child nutritional status in Ethiopia and Africa at large.

**Objective:** To assess the effectiveness of positive deviant approach to improve appropriate feeding practices and nutritional status in West Omo Zone, Maji District: A Cluster Randomized Trial

**Methods:** A Clustered randomized trial will be employed in Maji district. A multistage sampling strategy will be employed. Thirty-eight clusters will be selected from Maji districts (18 intervention arm and 18 control arm). Similar clusters will be paired together to form 18 pairs. For each of the 18 pairs, computer-generated randomization will be used to allocate clusters to intervention and control groups. The number of clusters and cluster size will be fixed. Equal numbers of families will be recruited from each cluster through systematic random sampling. A total of 516 mothers/caregivers with the infant and child aged from 0-24 will be included. In the intervention arms and the control arms have 258 each. The intervention arm receives 12 month positive deviance intervention. One positive deviance mother will be assigned for six non-deviant mothers and makes regular visits every 15 days and educates appropriate feeding practices based on the prepared manuals of infant and young child feeding. Data will be collected at baseline, midline (3 months) and endline (16 months) of the intervention. In the three data, collection time nutrition-related knowledge, attitude and self-efficacy, breastfeeding, child’s diet using 24-hour-based dietary recall questionnaire, and infant and young child feeding (IYCF) practices and anthropometric measures (weight and height) of the child will be elicited at baseline, midline, and endline. The data will be checked for distribution, missing values, and outliers, cleaned and analyzed using STATA 13 Software. Descriptive statistics, frequencies and means, and non-parametric values for improvement in nutritional status will be done. Linear standardized multiple regression modeling will be employed to compare means for improvement among the intervention arms. Both paired samples and independent-sample t-test and repeated measures of ANOVA will be done. For the experimental and control groups, χ2 tests and t-tests will be conducted for categorical and continuous measures, respectively. Multicollinearity will be checked using the variance inflation factor (VIF) before multivariable multilevel mixed-effects regression analysis. Variables with a p-value of less than ≤0.05 will be used as a cutoff point for statistical significance.

BACKGROUND/ STATE OF THE ART

The Global Strategy for infant and young child feeding (IYCF) provided a framework for action in 10 areas and identified the need to assess the implementation of policies and programs to identify gaps and to take action to bridge them (1). Primarily, malnutrition occurs during the first 2 years of life as children have a high demand for nutrients to support rapid growth and development; they are more susceptible to infection; they have heightened sensitivity to biological programming and are dependent on others for nutrition, care, and social interactions. Hence, the first 2 years of life is considered as a critical window of opportunity to address malnutrition. From all proven preventive health and nutrition interventions infant and young child feeding has the greatest potential impact on child survival (2).

Childcare is a complex concept including a range of behaviors and practices of caregivers that provide the food, healthcare, stimulation, and emotional support necessary for children’s healthy survival, growth and development (3). Adequate nutrition knowledge, positive attitudes and perceptions, and good practices of parents on infant and young child feeding, among others, are essential for the optimal growth of children. Hence, the involvement of both parents in IYCF is essential. The World Health Organization (WHO) and the United Nations International Children’s Fund (UNICEF) formulated the global strategy on IYCF to promote optimal breastfeeding and complementary feeding practices. Optimal breastfeeding, followed by the provision of safe and appropriate complementary foods introduced at the appropriate time (starting at six months), improves the growth and development of a child. Despite having the global strategy on IYCF in place, the statistics for breastfeeding and complementary feeding practices in the developing countries are a cause for concern. For instance, it is documented that only about 39% of infants in developing countries, and 25% in Africa, are exclusively breastfed in the first six months of life. Moreover, 6% of infants in developing countries are never breastfed (4).

Ethiopian government developed and implemented the Infant and Young Child Feeding (IYCF) guideline in 2004 to improve feeding practice. Based on the guideline IYCF messages were given at health institution and community level. However, the majority of mothers inappropriately fed their children (2). Breastfeeding and complementary feeding practices are underlying determinants of child development and nutrition). Though accepted indicators to assess breastfeeding practices have been in common use for nearly two decades, developing simple measures to assess complementary feeding practices has been more elusive. Child feeding is a complex concept that encompasses multiple dimensions including the diversity of the diet, the texture and nutrient density of complementary foods, the maintenance of exclusive and partial breastfeeding, and the manner and frequency with which foods are fed (5).

Ethiopia is also struggling to reduce infant mortality that results from malnutrition in a different part of the country. The Sekota Agreement, signed in July 2015, set a goal of eliminating stunting of infants under 2 years of age by 2030 and to this end, a 1,000 days Nutrition Service Programme was launched from October 2016 across the nation (6). Reduction of child morbidity and mortality can be reached only when IYCF practices are properly implemented to ensure adequate nutrition during the early childhood period. Despite strong evidence on the benefit of IYCF, inappropriate IYCF practices have been widely documented in low- and middle-income countries (10). Designing and implementing the parsimonious behavioral intervention like positive deviance (PD) approach in the resource-limited community found to be important in the community to address poor infant and young child feeding practices. The approach seeks out “positive deviants” in the community and uses their existing solutions to bring about sustainable behavioral and social change (7 ,8).

In past few years, many interventions have been conducted in Ethiopia much of which is related to information education communication and behavioral change communication focusing on the predisposing factors to improve infant and young child feeding practice which ultimately aimed to improve child nutrition and growth provided by health extension workers, health professional, and volunteers. However, we need to look other approaches which will have potential in catalyzing changes by community engagement. Hence, the proposed study is aimed to improve child nutrition influencing the health by causing undernutrition which has long-lasting effects among the children. Engaging the community to solve their problem on their own is particularly important in poor countries like Ethiopia. Therefore, we need to look into the positive deviant mothers solved their problems by themselves despite the resource limitations in poor communities. Therefore, this study is aimed to apply a positive deviant approach to improve infant and young child feeding practices in West Omo zone, Ethiopia which has a high prevalence of undernutrition. Hence the lesson obtained from this study will be diffused to other communities for the betterment and solving the nutrition-related problems in Ethiopia. Moreover, the lesson will help to provide the policymakers a future direction for nutrition-related intervention in Ethiopia.

HYPOTHESIS

Positive deviant approach can significantly improve appropriate feeding practices and nutritional status.

1. Postive deviant approach can significantly improve mothers’ self-efficacy, knowledge and attitude of appropriate feeding practices.
2. Postive deviant approach can significantly improve breastfeeding outcome among mothers.
3. Postive deviant approach can significantly improve dietary diversity and minimum meal frequency (complementary feeding) outcome among mothers.
4. Postive deviant approach can significantly improve infant and young child feeding practices
5. Postive deviant approach can significantly improve nutritional status (anthropometric measure) infant and child.

OBJECTIVES

Main Objective

The overall aim of this study is to evaluate the effectiveness of positive deviant approach to improve appropriate feeding practices and nutritional status in West Omo Zone, Maji District: A Cluster Randomized Trial

Specific objectives

1. To assess the effect of positive deviant approach in improving self-efficacy, knowledge, and attitude related to infant and young child feeding among the interventional group
2. To compare the effectiveness of positive deviant approach in improving exclusive breastfeeding (0 to 6 months) comparing intervention arms with no-intervention arms
3. To investigate the effectiveness of positive deviant approach in improving minimum dietary diversity and minimum meal frequency (complementary feeding) comparing the intervention arms with no-intervention arms.
4. To compare the effectiveness of positive deviant approach in IYCFP (0-24 months) among the intervention with no- intervention arms
5. To assess the effectiveness of positive deviant) approach in improving nutritional status (anthropometric measure) intervention arms with no intervention arms

INTERVENTIONS

**The intervention description**

**Control arm:** mothers in the control group will receive the routine health and nutrition education provided by health extension workers (HEWs) working in their kebeles and zones.

**Intervention arm:** mothers in the intervention arm will receive positive deviant intervention by selected positive deviant mothers. The intervention is composed of the following elements: a) breastfeeding education to raise knowledge, attitude, and breastfeeding self-efficacy, b) complementary feeding support, c) counseling on how to increase consistency, quantity, and frequency of foods, using locally available foods, and d) practical demonstration how to cook locally available food items, counseling, and support by positive deviant mothers.

After being trained, positive deviant mothers will provide infant and young feeding education and support to the selected non-positive deviant mothers. Besides the routine information and education provided to the mothers, each visit will be designated to cover a specific topic related to the outcome of the study.

**Nutrition education for selected mothers**

The intervention arm will receive IYCFP and nutrition education with standard manuals prepared using the local language. During each visit, positive deviant mothers will cover the details of the importance of breastfeeding, complementary feeding, and feeding an ill child. The discussion will combine the use of information education and communication (IEC) material and practical demonstration on proper child feeding (breastfeeding and complementary feeding). Mothers will be encouraged to ask any questions related to the topic discussed.

Positive deviant mothers will use culturally appropriate language in the form of a poster to illustrate the new information (eg. correct and incorrect breastfeeding, preparation of the enriched flour, appropriate consistency (thickness) and inappropriate consistency of complimentary food, the importance of significant others support) and the benefit of applying the recommended infant and young child feeding practices (pictures of the babies who were appropriately fed versus those who were not).

**Every fifteen days visits:** positive deviant mothers will visit non-positive deviant mothers every 15^th^ days, with each mother visited twice a month for education and demonstration. During each visit, mothers will be observed breastfeeding, appropriate feeding, and preparation for complementary feeding provided, solving any breastfeeding problems, inappropriate feeding, dietary diversity, required consistency, and hands-on guidance when necessary. They will support and encourage the mothers to follow the appropriate infant and young child feeding practice from 0-24 months. Positive deviant mothers will also promote personal and domestic hygiene, such as handwashing before feeding, after toilet, and changing babies' diapers.

Sample size

**Sample Size Determination**

The sample size was calculated using the sample size formula for cluster randomized control trials. The following formula was used to calculate sample size with the following assumption to improve appropriate IYCFP from 7% to 14 % (9), at 95% confidence interval (CI), 80 % power, assuming an intra-correlation coefficient (ICC) of detectable differences in IYCFP indicators in Ethiopia which is 0.03 (10) .

n= DE*(z_α/2_ + z_β_ )^2^ $\frac{p1\left( 1-p1 \right)+p2 (1-p2)}{\left( p1-p2 \right)2}$ , DE= 1+ ρ(m-1)

*Where:*

*p1: the proportion of outcome from the control group= 0.07, p2: the proportion of expected outcome from the intervention group =0.14,*$\alpha$ *:level of significant =0.05, Power (1-* *β)=0.8, Z-Alpha value (Z _α_*_/2_*)=1.96, Z-Beta value (z_β_)=0.84, ρ : intracluster correlation=0.03, m=number of mothers in each cluster =12 , DE: design effect, calculated using the following formula =* DE= 1+ *ρ(m-1)=1.33 and n=required sample size.* *Assuming the number of mothers in each cluster is 12.*

*n= 1.33 (1.96+0.84)^2^*  $\frac{\boldsymbol{0.07}\left( \boldsymbol{1-0.07} \right)\boldsymbol{+0.14 (1-0.14)}}{\left( \boldsymbol{0.07-0.17} \right)\boldsymbol{2}}$ **=** 430

Adding 20% of the sample size for lost to follow-up, the final sample size is 516 mothers (258 from the intervention arm and 258 from the control arm). For a cluster size of 12, it was calculated that we need 36 clusters/zones.

Data analysis

**Data prossessing and analysis**

EpiData version 3.1 will be used for double data entry, and Stata version 16 (StataCorp) will be used for consistency checks and statistical analysis. Descriptive statistics will be utilized to assess and summarize sociodemographic, socioeconomic, child health status, child morbidity , and child feeding. The data for continuous variables will be given as a mean and standard deviation, or median and range, whereas categorical variables will be reported as a frequency and percentage. The t-test and analysis of variance will be used to compare group means for primary and secondary outcome variables. The Chi-square test will be used to examine the categorical variables. Child nutrition outcomes will be computed and compared to the WHO 2006 growth standards (11).

The results of group comparisons will be reported as a risk ratio for binary outcomes, equivalent to 2-sided 95 percent confidence intervals and related p values. All p-values will be scaled to two decimal places, with values less than 0.01 reported as < 0.01. Adjusted analyses utilizing baseline variables will be done using multivariate logistic regression to assess the ongoing effect of important baseline features on outcomes. For time-dependent variables such as morbidity, the Kaplan-Meier survival analysis will be employed. The intention-to-treat analysis will be utilized, and the clustering effect (using study zone as random intercept to account for clustering of subjects by zones) will be addressed. All analyses will be done with a 95% confidence interval. The significance level will be assigned at a p-value < 0.05.

For the qualitative data, all in-depth interviews and focus groups will be audio-recorded, which will be translated into English before being transcribed verbatim and thematically analyzed in ATLAS-ti version 9.1 using Systematic Text Condensation, a descriptive and exploratory analytic method (12).

Study design and population

A cluster-randomized controlled single-blinded parallel groups, two arms, trial with 1:1 allocation ratio was designed to examine positive deviant intervention (PDI) provided for mothers’ to improve IYCFP, nutritional outcomes, knowledge, attitude, self-efficacy, and health-seeking behaviors of mothers’ toward their infants and young children in Maji Woreda, West Omo Zone, South West region, Ethiopia. This study design was chosen to avoid contamination among treatment groups. Clusters are zones, i.e. small administrative units found in Maji Woreda, West Omo ZoneSample size

REFERENCES

1. Gupta A, Holla R, Dadhich JP, Suri S, Trejos M, Chanetsa J. The status of policy and programmes on infant and young child feeding in 40 countries. Health Policy and planning. 2013 May 1;28(3):279-98.

2. Demilew YM. Factors associated with mothers’ knowledge on infant and young child feeding recommendation in slum areas of Bahir Dar City, Ethiopia: cross sectional study. BMC research notes. 2017 Dec;10(1):191.

3. Amugsi DA, Mittelmark MB, Lartey A, Matanda DJ, Urke HB. Influence of childcare practices on nutritional status of Ghanaian children: a regression analysis of the Ghana Demographic and Health Surveys. BMJ open. 2014 Nov 1;4(11):e005340..

4. Kumwenda W. *Parental and caregivers’ nutrition knowledge, attitudes, perceptions and practices on infant and young child feeding (aged zero to 24 months) in Mzimba-north district, Malawi* (Doctoral dissertation, University of Pretoria).

5. Jones A. Overcoming barriers to improving infant and young child feeding practices in the Bolivian Andes: the role of agriculture and rural livelihoods.

6. FDRo E. The 2017 voluntary national reviews on SDGs of Ethiopia: Government commitments, national ownership and performance trends.

7. Bullen PB. A multiple case study analysis of the positive deviance approach in community health.

8. Seonandan P, McKerrow NH. A review of infant and young child feeding practice in hospital and the home in KwaZulu-Natal Midlands. South African Journal of Clinical Nutrition. 2016 Sep 14;29(3):111-5.

9. Ethiopia Demographic and Health Survey. 2016.

10. Moss C, Bekele TH, Salasibew MM, Sturgess J, Ayana G, Kuche D, et al. Sustainable Undernutrition Reduction in Ethiopia ( SURE ) evaluation study : a protocol to evaluate impact , process and context of a large-scale integrated health and agriculture programme to improve complementary feeding in Ethiopia. 2018;1–11.

11. WHO Child Growth Standards. Dev Med Child Neurol. 2009;51(12):1002–1002.

12. Malterud K. Systematic text condensation: A strategy for qualitative analysis. Scand J Public Health. 2012;40(8):795–805.
