# Supplementary material for: Effectiveness of a positive deviance approach to improve appropriate feeding and nutritional status in South West Region, Ethiopia: A study protocol for a cluster randomized control trial
Source: PLoS One. 2024 Jan 2;19(1):e0266151. doi: 10.1371/journal.pone.0266151 (PMC10760826; doi:10.1371/journal.pone.0266151)
Supplement: S3 File — (DOCX) [file pone.0266151.s003.docx]

**World Health Organization Trial Registration Data Set.**

| **Data category** | **Information** |
| --- | --- |
| Primary registry and trial identifying number | **www.pactr.org** PACTR202108880303760 |
| Date of registration in primary registry | August 30, 2021 |
| Secondary identifying numbers | Not applicable |
| Source(s) of monetary or material support | Jimma University, Ethiopia |
| Primary sponsor | Jimma University, Ethiopia |
| Secondary sponsor(s) | Not applicable |
| Contact for public queries | *Jimma University,*  *Email:* [*juirb@gmail.com*](mailto:juirb@gmail.com)  *AA*    *Cancer Screening Unit*, +34932607959, [prevenciocolon@iconcologia.net](mailto:prevenciocolon@iconcologia.net)  ] |
| Contact for scientific queries | Abraham Tammirat, Ph.D student, phone number :+251-911722420, Email: [abraham.tamirat@ju.edu.et](mailto:abraham.tamirat@ju.edu.et) |
| Public title | Effectiveness of positive deviant (hearth nutrition education) intervention to improve appropriate feeding practices and nutritional outcomes in West Omo Zone, Maji District: Southwest Ethiopia: A cluster randomized control tria |
| Scientific title | Effectiveness of positive deviant (hearth nutrition education) intervention to improve appropriate feeding practices and nutritional outcomes |
| Countries of recruitment | Ethiopia |
| Health condition(s) or problem(s) studied | Infant and young child feeding practices (nutrition) |
| Intervention(s) | Active comparator: positive deviant (hearth nutrition education) interventions: 1) improvement in infant and young child feeding practices, 2) change in knowledge, attitude and breastfeeding self-efficacy, 3) infant growth  Placebo comparator: Usual Care: Health education provided by health professionals. |
| Key inclusion and exclusion criteria | Inclusion criteria: infants and young children (IYC) age 0-24 month. Inclusion criteria will be mothers living in the selected clusters with no plan to move away during the intervention period, without psychiatric illness, capable of giving informed consent, and willing to be visited by supervisors and data collectors. Infants and young children with no severe malnutrition and no severe illness will be included.  Exclusion criteria will be mothers with a severe psychological illness which will interfere with consent and children with severe illness or clinical complications, which will potentially influence the study outcomes will excluded |
| Study type | Interventional Allocation: randomized Intervention model: parallel assignment Masking: Open Label ( Primary purpose: Health Services Research |
| Date of first enrolment | January 2022 |
| Target sample size | 516 (anticipated) |
| Recruitment status | Not yet recruiting |
| Primary outcome(s) | Intervention 1:Breastfeeding knowledge, attitude, and self-efficacy  Intervention 2: Complementary feeding knowldge, attitude and self-efficacy  Intervention 3 Infant growth |
| Key secondary outcomes | Barriers and facilitatorsof IYCFP and morbidity status |
