# Supplementary material for: Effectiveness of a positive deviance approach to improve appropriate feeding and nutritional status in South West Region, Ethiopia: A study protocol for a cluster randomized control trial
Source: PLoS One. 2024 Jan 2;19(1):e0266151. doi: 10.1371/journal.pone.0266151 (PMC10760826; doi:10.1371/journal.pone.0266151)
Supplement: S4 File — (DOCX) [file pone.0266151.s004.docx]

**Participant Information and Consent Form for Baseline Interview**

**Participant ID**

**Annex 3: English version information sheet**

**Title of the research Project**: Effectiveness of a positive deviance approach to improve appropriate feeding and nutritional outcomes in South West Region, Ethiopia: A study protocol for a cluster randomized control trial

**Name of Investigator:** Abraham Tamirat (PhD fellow)

**Name of the Organization**: Jimma University, Institute of health, Faculty of Public Health, Department of Health, Behavior and Society

**Introduction:** This information sheet is prepared to Effectiveness of a positive deviance approach to improve appropriate feeding and nutritional outcomes in South West Region, Ethiopia: A study protocol for a cluster randomized control trial

Hello! How are you? My name is __________. Now I am a research team member to be conducted here by a post graduate student in institute of health in Jimma University, supervised by assigned supervisor. The purposes of the study is assessing Effectiveness of positive deviant (hearth nutrition education) approach to improve appropriate feeding practices and nutritional status in West Omo Zone, Maji District: A Cluster Randomized Trial

**Procedure / what the study involves**

The study may be advantageous in identifying barriers to appropriate feeding among infant and young child. Therefore, addressing the barriers through positive deviant mothers to improve the nutritional status is the ultimate goal of the study.

**Confidentiality**

All the genuine information obtained from you will be strictly kept confidential, your participation is surely voluntary, and no monetary incentives will be given for your participation in the study. You can withdraw any time during conducting the study, also your participation, non-participation, or refusal to answer questions will not have any effect on your life, and your name will not be recorded on this form. Risk and /or Discomfort: Since the study will be conducted by taking appropriate information , it will not inflict any harm on you and the community.

The name or any other identifying information will not be recorded on the checklist and all information taken from the individual will be kept strictly confidential and in a safe place. The information retrieved will only be used for the study purpose there will be no other third party accessing the information provided. After the study has been conducted the questionnaire will be burnt out. Benefits: The researchers have no direct benefit for one whose document/ record is included in this research.

**Risks**

You may be uncomfortable with some of the questions that I will ask. You are perfectly entitled to refuse to discuss issues that you do not want to.

**Benefits**

There are no direct benefits to you in your taking part in this interview. However, what we learn from this study would help the Ministry of Health to make important decisions regarding how to improve infant and young child feeding.

**Compensation**

You will not receive payment for participating in the study.

**Contact details**

If you have any question Mr. Abraham Tamirat is the contact person. Can be reached through a call at +251-911722420

**Consent Declaration**

If you agree to voluntarily participate in the study, please sign or write your initial or your thumb print below to show that you understand the information above and that your consent is given voluntarily.

|  |  | Participant  Initials / thumb print |
| --- | --- | --- |
| 1 | I have received and read or had read to me the information sheet: Participant Information and Consent Form for Baseline Interview -provided by the Researcher that explains in detail the reasons for the study. | --------------------------- |
| 2 | I have understood the purpose of the research. | --------------------------- |
| 3 | I have asked all the questions that I have about the purpose of the research and feel that I have enough information about it. | --------------------------- |
| 4 | I understand the reasons for this study. | --------------------------- |
| 5 | I am willing to take part in the study. | --------------------------- |
| 6 | I understand what I will be required to do if I participate in the study. | --------------------------- |
| 7 | I know that I have the right to leave the study at any time or to refuse to answer any questions. | --------------------------- |
| 8 | If I do not agree to take part in this study, I understand that I will not be penalized for doing so by the researcher nor by any medical service providers in the future. | --------------------------- |
| **9** | **I voluntarily agree to take part in this study** | --------------------------- |

------------------------------------------------- -----/-------/-------- -----------------------------------

Participant name Date Signature or thumb print

**If the participant gave verbal consent, please enter the name of person who witnessed the consent here, and their signature:**

------------------------------------------------- -----/-------/-------- -----------------------------------

Name of Witness (BLOCK CAPITALS) Date Signature or thumb print

------------------------------------------------- -----/-------/-------- -----------------------------------

Name of person obtaining consent Date Signature
