# Supplementary material for: Effectiveness of a positive deviance approach to improve appropriate feeding and nutritional status in South West Region, Ethiopia: A study protocol for a cluster randomized control trial
Source: PLoS One. 2024 Jan 2;19(1):e0266151. doi: 10.1371/journal.pone.0266151 (PMC10760826; doi:10.1371/journal.pone.0266151)
Supplement: Attachment — Submitted filename: Comments Plos one.docx [file pone.0266151.s005.docx]

**Comments**

The study protocol by Gizaw et al., is one of a kind which describes the effectiveness of a positive deviant intervention to improve appropriate feeding practices and nutritional outcomes in West Omo Zone, Maji District Ethiopia. But there are some revisions to be made in the paper, pertaining to which the manuscript would be suitable for publication:

**Abstract:**

*Comment 1:* The author used the word inclusion criteria in the abstract. A brief sentence can be added about how the author selected the paricipants based on inclusion and exclusion criteria.

**Introduction:**

*Comment 2:* Does the introduction provide sufficient background information for readers not in the immediate field to understand the problem/hypotheses?

The introduction provides a good, generalized background of the topic that quickly gives the reader an appreciation of the positive deviant to improve appropriate feeding practices and nutritional outcomes. However, to make the introduction more substantial, the author may wish to provide several references to substantiate the claim made in the second paragraph (that is, provide references to other groups who do or have done research in this area).

**Methods**

*Comment 3*: In the methods, a sentence could be added on the pilot survey after which the questionnaire and tools could be modified.

*Comment 4*: Before data collection among participants, how the reliability and validity of the translated questionnaire was checked?

**Discussion**

*Comment 5:* Limitations of the study could be added.
