# Supplementary material for: Effectiveness of a positive deviance approach to improve appropriate feeding and nutritional status in South West Region, Ethiopia: A study protocol for a cluster randomized control trial
Source: PLoS One. 2024 Jan 2;19(1):e0266151. doi: 10.1371/journal.pone.0266151 (PMC10760826; doi:10.1371/journal.pone.0266151)
Supplement: Attachment — Submitted filename: response to the editor-2.docx [file pone.0266151.s006.docx]

**Dr. Sourabh Dutta, PLOS ONE	Date: 14/08/2022**

*Academic Editor*

Effectiveness of a positive deviance approach to improve appropriate feeding and nutritional outcomes in South West Region, Ethiopia: A study protocol for a cluster randomized control trial: PONE-D-22-07220

We thank the Senior Editor as well as the reviewers for their comments and appreciate the opportunity to submit a revised manuscript. The manuscript has been revised in accordance with the Editor’s comments and suggestions. Our specific response to each comment is detailed below, including all of the corresponding revisions in the manuscript and supplement (pages and paragraphs correspond to the “All Markup” format in Microsoft Word).

| **Editor Comments** | **Response** | **Page Number** |
| --- | --- | --- |
| 1. We notice that your manuscript file was uploaded on March 14, 2022. Please can you upload the latest version of your revised manuscript as the main article file, ensuring that does not contain any tracked changes or highlighting.  This will be used in the production process if your manuscript is accepted. Please follow this link for more information: [http://blogs.PLOS.org/everyone/2011/05/10/how-to-submit-your-revised-manuscript/](http://blogs.plos.org/everyone/2011/05/10/how-to-submit-your-revised-manuscript/) | We have separated the main manuscript without changes in the file name of  “ATG-Main Manuscript revised Trial A1922” | N/A |
| 2. Thank you for submitting the above manuscript to PLOS ONE. During our internal evaluation of the manuscript, we found significant text overlap between your submission and the following previously published works, some of which you are an author.  -   <https://link.springer.com/article/10.1186/s12887-018-1278-5>  We would like to make you aware that copying extracts from previous publications, especially outside the methods section, word-for-word is unacceptable. In addition, the reproduction of text from published reports has implications for the copyright that may apply to the publications.  Please revise the manuscript to rephrase the duplicated text, cite your sources, and provide details as to how the current manuscript advances on previous work. Please note that further consideration is dependent on the submission of a manuscript that addresses these concerns about the overlap in text with published work.  We will carefully review your manuscript upon resubmission, so please ensure that your revision is thorough. | We have made all the significant changes, definitely paraphrased all the similar phrases and paragraphs. | N/A |
| We note that the grant information you provided in the ‘Funding Information’ and ‘Financial Disclosure’ sections do not match. When you resubmit, please ensure that you provide the correct grant numbers for the awards you received for your study in the ‘Funding Information’ section. | We request excuse for the fund number and information provided. The grant that we perceived to be secured from Jimma University, is not yet secured. Therefore, there is no funding information that we can provide. We have secured only ethical letter from Jimma University. | N/A |
| Your ethics statement should only appear in the Methods section of your manuscript. If your ethics statement is written in any section besides the Methods, please move it to the Methods section and delete it from any other section. Please ensure that your ethics statement is included in your manuscript, as the ethics statement entered into the online submission form will not be published alongside your manuscript. | We have added it to the method part. | Page 17 |
| Please ensure that you refer to Figure 3 in your text as, if accepted, production will need this reference to link the reader to the figure | We have added it wrongly and removed it from the lists of figures. | **N/A** |
