# Supplementary material for: Effectiveness of a positive deviance approach to improve appropriate feeding and nutritional status in South West Region, Ethiopia: A study protocol for a cluster randomized control trial
Source: PLoS One. 2024 Jan 2;19(1):e0266151. doi: 10.1371/journal.pone.0266151 (PMC10760826; doi:10.1371/journal.pone.0266151)
Supplement: Attachment — Submitted filename: Response to the editor R19.docx [file pone.0266151.s007.docx]

**To:** Dr. Jianhong Zhou
Staff Editor
PLOS ONE
Title of the manuscript : " **Effectiveness of a positive deviance approach to improve appropriate feeding and nutritional status in South West Region, Ethiopia: A study protocol for a cluster randomized control trial**"
We thank the senior editor for their comments and appreciate the opportunity to submit a revised manuscript. The required documents were attached in accordance with the comments and suggestions.

| **Comment of the editor** | **Response** | **Page Number** |
| --- | --- | --- |
| Please review your reference list to ensure that it is complete and correct. If you have cited papers that have been retracted, please include the rationale for doing so in the manuscript text, or remove these references and replace them with relevant current references. Any changes to the reference list should be mentioned in the rebuttal letter that accompanies your revised manuscript. If you need to cite a retracted article, indicate the article’s retracted status in the References list and also include a citation and full reference for the retraction notice. | We have addressed the comment on the manuscript. We have excluded the retracted references, corrected cross references and used updated possible as much as possible. However, some references are very important which depicted the steps of conducting positive deviant approach and we haven’t found updated references and we used it as it is. | 26,27 & 28 |
| As reviewer 2 and reviewer 3 have hinted, the manuscript is rather confusing to follow. There are fundamental problems in understanding what exactly is the definition of "a positive deviant mother”. | We have included in the method section of the manuscript | 11 |
| As per page 6, the NON-positive deviant mothers should have a infant/child aged 0-24 months, the child's Height-for-age Z (HAZ) scores HAZ < −2 and a child with NO severe malnutrition will be included in the study. Presumably <-2 z score means worse than -2. It is not clear from "a child with no severe malnutrition" whether this refers to the index child who is expected to be stunted or a sibling of the child. If it is the index child, then is the child supposed to have no severe malnutrition but be stunted at the same time, and if so, why? | We appreciate the comment that the editor trying to raise. It is quite confusing and we have addressed the comment in the “eligibility criteria for the participants” section of the manuscript. | 8 |
| On page 9, the POSITIVE deviant mothers are defined as those who have a number of positive attributes but their child should apparently have the following attributes which are all negative "was a big baby who is losing weight now", "have any severely malnourished sibling", "have any serious or typical social or health problem", "have families enrolled in a supplementary feeding program". | We have addressed the comment in the “intervention” section of the manuscript. We totally edited the section. | 11 |
| Apart from the ambiguity about the definition of positive deviant and nonpositive deviant mothers, the manuscript lacks clarity in many places. | We have addressed the comment in the “intervention” section of the manuscript. We tried to maintain clarity throughout the manuscript by editing the language errors and grammar errors. | 11 |
| The authors must take the help of a colleague whose primary language is English. There are many sentences in the manuscript that are challenging to understand. Perhaps the authors were not able to get their point across clearly because of a language barrier. | In addition to the corresponding authors, one of the co-author from USA took full responsibility in editing the language throughout the manuscript . | N/A |
| Reviewer #1: The previous reviewer's comments have been addressed. The methodology has been strengthened and strengths and limitations of the stud have been added. | No comments are provided and we appreciate for being acting as a reviewer of our manuscript. | N/A |
| **Reviewer 3 comments** | | |
| Comment 1: The study design is stated to be a single blinded RCT . It is stated that because of the nature of intervention, mothers cannot be blinded but the intervention assignment is concealed from the interviewers collecting outcome data. But how will it be possible to conceal the intervention data from the interviewers as they are collecting data from mother herself who knows it? How is blinding possible in such a situation? | Dear reviewer thanks for raising most important concern. However, concealing the intervention group from the interviewer are most important for assuring and understanding the effect of the intervention (PDA). This potentially minimize the bias of the data collection. This can be done by recruiting the data collectors who are away or living out of the intervention area. The baseline, midline and endline data collection will be conducted simultaneously both to the intervention and control groups, where the PI and Co-PI know, but the data collectors don’t know. The cluster will be coded (01-36) and hh-labelling (001-516) will be given. The research team knows which cluster is the intervention and control group at the end of the data collection. Also the study hypothesis also concealed from health extension workers and other volunteers working on health because once they know it they will bias the outcome by providing addition lesson on IYCFP to routine health education. This will bias the effect of the PDA. | 9 |
| Comment 2: Regarding identification of positive deviant mother , the criteria from child’s side is stated to be a big baby who is losing weight now , have any severely malnourished sibling , have any social or health problem are taken as babies who will be considered. But should it not be other way round? Their families should not be considered for positive deviance. | We have addressed the comment in the “intervention” section of the manuscript. We totally amended the section. | 11 |
| Comment 3: Though the authors tried to mention that every fortnightly positive deviant mothers will visit non positive deviant mothers, the practical aspects like how the they would be transported , the logistics , time taken to do so , how they are going to help other mothers need to be addressed. | We have addressed the comment in the “Intervention activities” section of the manuscript. We totally amended the section. | 13 & 14 |
| **Thank you for the important comments!** | | |
